# Depressive symptoms post hip fracture in older adults are associated with phenotypic and functional alterations in T cells

**DOI:** 10.1186/s12979-014-0025-5

**Published:** 2014-12-16

**Authors:** Niharika Arora Duggal, Jane Upton, Anna C Phillips, Peter Hampson, Janet M Lord

**Affiliations:** School of Immunity and Infection, University of Birmingham, Birmingham, B15 2TT UK; MRC-Arthritis Research UK Centre for Musculoskeletal Ageing Research and, University of Birmingham, Birmingham, B15 2TT UK; School of Sport, Exercise and Rehabilitation Sciences, University of Birmingham, Birmingham, B15 2TT UK

**Keywords:** Depressive symptoms, Hip fracture, T cell, Stress, Immunity, Inflammation, Ageing, Cortisol

## Abstract

**Background:**

Ageing is accompanied by reduced immunity, termed immunesenescence. The immune system does not act in isolation and is sensitive to both psychological and physical stress. Hip fracture is a common physical stressor in older adults with a high incidence of new onset depression, which relates to poorer prognosis. We therefore set out to examine the possible synergistic effects of physical stress (hip fracture) and psychological stress (depressive symptoms) on the aged immune system.

**Results:**

T cell phenotype and function was assessed in 101 hip fracture patients (81 female) 6 weeks after hip fracture and 43 healthy age-matched controls (26 female). 38 fracture patients had depressive symptoms at 6 weeks. T cell frequency (p = .01) and numbers (p = .003) were both lower in depressed hip fracture patients compared to healthy controls. The frequency of senescent CD28^-ve^ (p = .001), CD57^+ve^ (p = .001), KLRG1^+ve^ (p = .03) CD8 T cells, as well as senescent CD28^-ve^ CD4^+ve^ (p = .01) and CD57^+ve^ CD4^+ve^ (p = .003) T cells were higher in depressed hip fracture patients compared with healthy controls and the frequency of CD28^-ve^ CD8 T cells was also higher when compared to patients with hip fracture alone (p = .01). Additionally, activated CD69^+ve^ (p = .005) and HLADR^+ve^ (p < .001) CD8 T cells, were also higher in depressed hip fracture patients compared to healthy controls. On examining cytokine production by activated T cells, a significant increase in TNFα (p = .03) and IL6 (p = .04) production was observed in CD4 T cells from hip fracture patients with depressive symptoms compared to healthy controls.

**Conclusions:**

As none of the patients in the study had a prior history of depression, our data suggest that the development of depressive symptoms in hip fracture patients is associated with altered T cell phenotype and increased pro-inflammatory function which is not seen in patients who do not develop depression after hip fracture. Treating depressive symptoms promptly in hip fracture patients may therefore improve immunity and outcomes in these patients.

**Electronic supplementary material:**

The online version of this article (doi:10.1186/s12979-014-0025-5) contains supplementary material, which is available to authorized users.

## Background

Healthy ageing is associated with a significant decline in immune competence and ability to mount a robust immune response, termed immunesenescence [1]. This compromised immune response is due in large part to extensive remodelling of the adaptive immune system including thymic atrophy [2], a decrease in the CD4:CD8 ratio [3,4] and an increase in the naïve: memory T cell ratio [4,5]. Immune ageing also includes marked phenotypic alterations in T cells associated with functional senescence, notably loss of the co-stimulatory receptor CD28 and acquisition of receptors normally associated with Natural Killer cells, for example KLRG-1 [6]. The result is an accumulation with age of CD28^-ve^, KLRG-1^+ve^, CD57^+ve^ senescent T cells [7,8] as well as activated HLADR^+ve^ T cells [9]. Furthermore, ageing is accompanied by a decline in CD4 helper activity [10] and a shift from a Th1 (IFNγ, IL2) to Th2 (IL10, IL4) phenotype on activation [11], but an increase in pro-inflammatory cytokine production by T cells overall [12,13].

Healthy older individuals have been reported to experience greater levels of stress, anxiousness and depression than young adults [14]. Meta-analysis of the literature over the past few decades has led to the development of the hypothesis that chronic stressful events are suppressors of immune function [15]. Interestingly, there is accumulating evidence suggesting that the effects of stress and age are interactive with chronic stress exacerbating the effects of ageing on immune function [16]. Our own work has shown that innate immunity is susceptible to the effects of stress, with neutrophil superoxide generation reduced in old hip fracture patients [17] and bereaved older adults [18].

Hip fracture is a common and potentially devastating injury in older adults [19]. 1 in 3 older adults fall each year and it is predicted that this will result in 117,000 hip fractures by 2016 [20]. Even though hip fracture is treatable, it is a severe physical stressor for older individuals. Medical events in older adults have been associated with a significant risk of developing depression [21] and a high rate of depression, ranging from 9 - 47%, has been reported in UK and US based studies of older adults with hip fracture [22]. Importantly, depression is associated with increased risk of infections and poor survival [23], impaired recovery and a reduced ability to regain pre-fracture levels of physical functioning [24].

The hypothalamus-pituitary-adrenal (HPA) axis acts as a pivotal regulator of the stress responses by mobilising energy reserves and modulating immune responses [25]. Glucocorticoids (GCs) are key effectors of the HPA axis and are potent immune suppressors. Dehydroepiandrosterone sulphate (DHEAS), a major steroid produced by the adrenal gland, has been reported to have anti-depressive, anti-glucocorticoid and immune-enhancing properties [26]. Some previous studies have suggested that healthy ageing is accompanied by hyperactivation of the HPA axis, especially in situations of chronic stress, resulting in prolonged exposure to cortisol [27]. This is due also in large part to dramatic changes in the serum level of DHEAS which reaches peak concentrations during the third decade of life, after which a steady decline occurs with age (1–2% per year); such that by the age of 80, DHEAS levels have reached 10-20% of their peak level [28]. Therefore, the current literature suggests that ageing is accompanied by an elevated cortisol:DHEAS ratio which may be a key factor contributing towards age associated immune dysregulation [29] and which might be heightened by chronic stress. In a previous study, our group reported a raised cortisol: DHEAS ratio in old hip fracture patients compared to comparable young trauma patients [30]. We have also reported recently that suppression of innate immunity, specifically neutrophil function, only occurred in those hip fracture patients that developed depressive symptoms [31].

The present study sought to test the hypothesis that psychological distress, specifically depressive symptoms, would act additively with the physical stress of hip fracture to amplify the effect of ageing upon adaptive immunity, with specific reference to T cells. It also examined the role of the cortisol and pro- and anti-inflammatory cytokines as potential mediators of any effects observed.

## Results

### Participant demographics

The full demographic profiles of the participants have been reported previously [31]. Patients were classified into two groups on the basis of their GDS scores: hip fracture patients with a GDS score of 5 or less were classified as non-depressed (HF; hip fracture only), those with a score of 6 or greater were categorised as having depressive symptoms (HF + D; Hip fracture patients with depressive symptoms). In this study we observed that 38 (37%) of the hip fracture patients had depressive symptoms 4–6 weeks after their fracture.

### Peripheral T cell numbers in hip fracture patients

**Figure 1:**
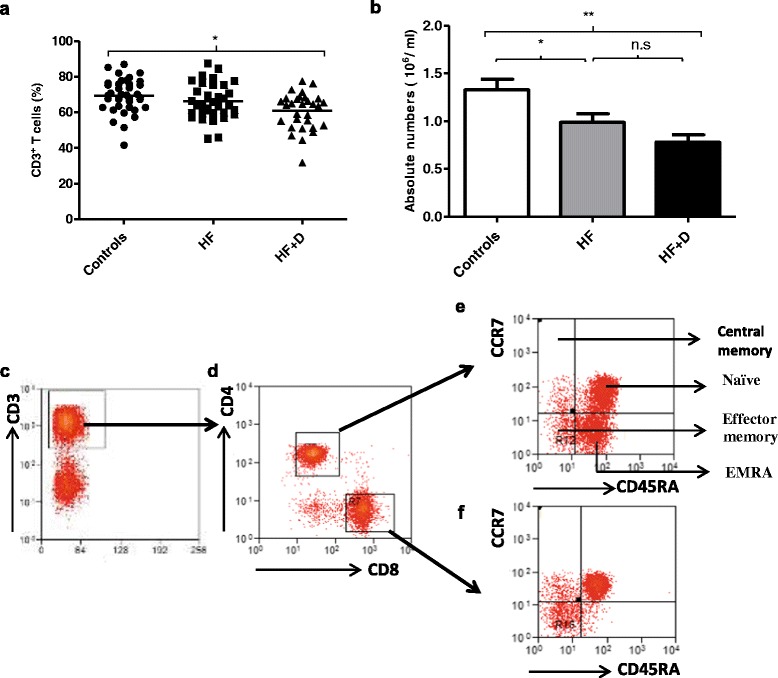
**Frequency of peripheral T lymphocytes in hip fracture patients with and without depressive symptoms versus healthy controls. (a)** The percentage of CD3^+ve^ T cells in healthy controls: n = 37; hip fracture patients without depression (HF): n = 38 and hip fracture patients with depressive symptoms (HF + D): n = 29.The solid bar represents the mean value. **(b)** The mean absolute number of T cells in healthy controls: n = 20; hip fracture patients without depression (HF): n = 19 and hip fracture patients with depressive symptoms (HF + D): n = 15. Data are mean ± SEM. *p < .05 and **p < .01. Representative flow cytometry plots and gating strategy of PBMCs from a young individual stained with anti-CD3 and **(c)** anti-CD4, and CD8, **(d)** and anti-CD45RA and -CCR7 **(e and f)**.

Peripheral T cells counts in hip fracture patients with and without depressive symptoms were compared with healthy older adults. Significant differences in percentages of CD3^+ve^ T cells in the PBMC pool were found between the three groups, F (2, 101) = 4.59, p = .01, η^2^ = .08, [Figure 1a], which was driven by a significant decline in percentage of T cells in hip fracture patients with depressive symptoms compared with healthy controls (p = .01) but not with hip fracture patients without depressive symptoms (p = .12). Similarly, on examining the absolute numbers of T cells in peripheral blood, significant differences were seen between our three groups, F (2, 51) = 7.18, p = .002, η^2^ = .22 [Figure 1b], with a decline shown in T cells in hip fracture patients with depressive symptoms compared with healthy controls (p = .003). When the above analyses were repeated with adjustment for age, sex and BMI, the results remained significant (data not shown).

### CD4:CD8 T cell ratio in hip fracture patients

On examining the percentage of CD4 T cells, no significant differences were observed between our three groups, F (2, 85) = .68, p = .50, η^2^ = .01 [Table 1]. However, significant differences occurred in absolute numbers of CD4 T cells, F (2, 52) = 6.01, p = .004, η^2^ = .18, driven by a significant decline in absolute numbers of CD4 T cells in hip fracture patients with depressive symptoms compared with healthy controls (p = .006) only. No significant differences were observed between the percentage of CD8 T cells, F (2, 97) = .13, p = .87, η^2^ = .003, or in absolute numbers of CD8 T cells between our three groups, F (2, 52) = 1.31, p = .27, η^2^ = .04, [Table 1]. Thus no significant differences were seen in the CD4:CD8 ratio between the three groups, F (2, 97) = 1.11, p = .33, η^2^ = .02 [Table 1].

**Table 1:** **Participant peripheral distribution of T cells by group**

**Variable**	**Controls**	**Mean (SD)**		
		**HF**	**HF + D**	**p**
CD4^+ve^ T cells (%)	65.55 (10.57)	68.97 (12.21)	67.40 (13.76)	.50
Absolute numbers (10^6^/ml)	.85 (.26)	.63 (.29)	.55 (.22)	.004
CD8^+ve^ T cells (%)	28.31 (10.01)	26.78 (12.23)	27.32 (14.34)	.87
Absolute numbers (10^6^/ml)	.32 (.22)	.25 (.16)	.21 (.18)	.27
CD4:CD8 ratio	2.80 (1.76)	3.41 (2.98)	3.56 (2.61)	.33

### Memory: naive T cell ratio in hip fracture patients

CD4 T cells and CD8 T cells can be divided into four differentiation subsets on the basis of expression of CD45RA and CCR7: naïve, central memory (CM), effector memory (EM) and terminally differentiated effector memory cells (EMRA) [32]. Figures 1c-f show representative FACs plots for PBMCs stained with anti-CD3 and anti-CD4, CD8, CD45RA and CCR7 antibodies to identify these subsets. On examining the percentage, F (2, 85) = .13, p = .87, η^2^ = .003, and absolute numbers of naïve CD4 T cells in peripheral blood, F (2, 51) = 2.07, p = .13, η^2^ = .07 no significant differences were found between subject groups [Table 2]. Further, on examining central memory CD4 T cell subsets, no significant differences occurred in their percentages, F (2,86) = .90, p = .40, η^2^ = .02, or absolute numbers F (2, 51) = 1.37, p = .26, η^2^ = .05, between the three groups [Table 2]. Similarly, no significant differences were found in the percentage, F (2, 86) = .20, p = .81, η^2^ = .005, or absolute numbers, F (2, 51) = .77, p = .46, η^2^ = .03, of effector memory CD4 T cells [Table 2]. Finally, no significant differences were seen in the percentage, F (2, 86) = .23, p = .79, η^2^ = .005, or in absolute numbers of effector memory RA CD4 T cells, F (2, 51) = 1.83, p = .16, η^2^ = .06 [Table 2]. Thus, the naïve:memory ratio for CD4 T cells also did not significantly differ between hip fracture patients with and without depressive symptoms and healthy controls, F (2, 89) = .21, p = .80, η^2^ = .005 [Table 2].

**Table 2:** **Naive and memory CD4 T lymphocytes in hip fracture patients**

**Variable**	**Controls**	**Mean (SD)**		
		**HF**	**HF + D**	**p**
Naïve CD4 T cells (%)	38.92 (19.65)	36.87 (20.55)	36.55 (18.11)	.87
Absolute numbers (10^6^/ml)	.35 (.27)	.22 (.22)	.21 (.15)	.13
CM CD4 T cells (%)	3.49 (6.99)	3.49 (3.37)	5.38 (6.98)	.40
Absolute numbers (10^6^/ml)	.01 (.01)	.03 (.02)	.02 (.02)	.26
EM CD4 T cells (%)	19.38 (10.62)	20.33 (10.93)	21.39 (15.31)	.81
Absolute numbers (10^6^/ml)	.14 (.07)	.14 (.11)	.10 (.08)	.46
EMRA CD4 T cells (%)	38.40 (17.15)	40.10 (17.36)	36.96 (16.42)	.79
Absolute numbers (10^6^/ml)	.31 (.14)	.26 (.17)	.22 (.10)	.16
Total memory CD4 T cells (%)	61.07 (19.64)	63.16 (20.54)	63.44 (18.04)	.87
Absolute numbers (10^6^/ml)	.46 (.14)	.39 (.21)	.34 (.15)	.12
Naive: memory ratio	.82 (.87)	.87 (.84)	.73 (.63)	.80

We then examined the distribution of naïve and memory cells amongst CD8 T cells. Firstly, no differences were observed in the percentage, F (2, 85) = .05, p = .94, η^2^ = .001, or absolute numbers of naïve CD8 T cells F (2, 51) = 1.77, p = .18, η^2^ = .06, between the three groups [Table 3]. Further, on examining memory CD8 T cell subsets, no significant differences were observed in the percentage, F (2,85) = 1.34, p = .26, η^2^ = .03, or absolute numbers F (2, 51) = .80, p = .45, η^2^ = .03, of central memory CD8 T cells between the three groups [Table 3]. Similarly, no significant differences were found in the percentage, F (2, 85) = 2.77, p = .07, η^2^ = .06, or absolute numbers of effector memory CD8 T cells, F (2, 51) = 1.21, p = .30, η^2^ = .04 [Table 3] and the percentage, F (2, 85) = .55, p = .57, η^2^ = .01, and absolute numbers of effector memory RA CD8 T cells, F (2, 51) = .50, p = .60, η^2^ = .01, did not differ between groups [Table 3]. Thus, the naïve: memory ratio for CD8 T cells did not significantly differ between the three groups, F (2, 86) = .12, p = .89, η^2^ = .003, [Table 3].

**Table 3:** **Naive and memory CD8 T lymphocytes in hip fracture patients**

**Variable**	**Controls**	**Mean (SD)**		
		**HF**	**HF + D**	**p**
Naïve CD8 T cells (%)	16.98 (16.12)	15.73 (13.11)	16.61 (13.89)	.94
Absolute numbers (10^6^/ml)	.05 (.07)	.02 (.04)	.02 (.01)	.18
CM CD8 T cells (%)	26.55 (14.61)	33.17 (20.09)	32.03 (16.01)	.26
Absolute numbers (10^6^/ml)	.06 (.06)	.10 (.11)	.09 (.09)	.45
EM CD8 T cells (%)	41.04 (13.27)	33.40 (16.16)	33.56 (14.44)	.07
Absolute numbers (10^6^/ml)	.13 (.09)	.09 (.10)	.09 (.08)	.30
EMRA CD8 T cells (%)	14.44 (11.09)	17.02 (13.50)	17.63 (13.64)	.57
Absolute numbers (10^6^/ml)	.04 (.04)	.04 (.09)	.02 (.02)	.60
Total memory CD8 T cells (%)	83.01 (16.12)	83.99 (12.89)	82.43 (14.65)	.92
Absolute numbers (10^6^/ml)	.27 (.20)	.21 (.16)	.19 (.19)	.39
Naive: memory ratio	.26 (.41)	.23 (.26)	.28 (.45)	.89

### Accumulation of senescent T cells in hip fracture patients with depressive symptoms

**Figure 2:**
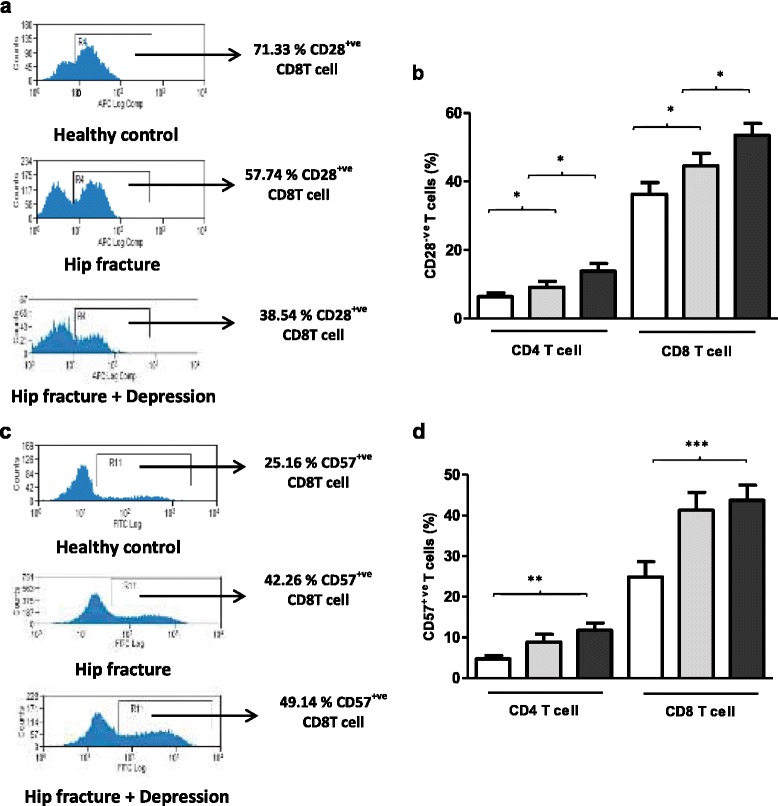
**Frequency of peripheral senescent T lymphocytes in hip fracture patients with and without depressive symptoms versus healthy controls. (a)** Representative flow cytometry plots showing frequency of CD28^+ve^ CD8 T cells in a healthy control, hip fracture and a hip fracture patient with depressive symptoms. **(b)** The percentage of CD28^-ve^ CD4 and CD8 T cells in healthy controls: n = 23 (open bars); hip fracture patients without depression n = 19 (grey bars); and hip fracture patients with depressive symptoms (black bars): n = 23. Data are mean ± SEM. *p < .05 and **p < .01. **(c)** Representative flow cytometry plots showing frequency of CD57^+ve^ CD8 T cells in a healthy control, hip fracture and a hip fracture patient with depressive symptoms. **(d)** The percentage of CD57^+ve^ CD4 and CD8 T cells in healthy controls: n = 23 (open bars); hip fracture patients without depression n = 19 (grey bars); and hip fracture patients with depressive symptoms (black bars): n = 23.

CD28 is a co-stimulatory molecule involved in T cell activation which is lost as T cells progress towards senescence. PBMCs were stained with an anti-CD28 antibody to identify CD28^-ve^ and CD28^+ve^ cells [Figure 2a]. On examining the frequency of circulating CD28^-ve^ CD4 T cells, we found significant differences between the three groups, F (2, 62) = 5.26, p = .008, η^2^ = .14, driven by a significant increase in the percentage of CD28^-ve^ CD4 T cells in hip fracture patients with depressive symptoms compared with healthy controls (p = .01) and hip fracture patients without depressive symptoms (p = .04) [Figure 2b]. Similarly, differences in the frequency of circulating CD28^-ve^ CD8 T cells were found between the three groups, F (2, 62) = 8.30, p = .001, η^2^ = .21, driven by a significant increase in the percentage of CD28^-ve^ CD8 T cells in hip fracture patients with depressive symptoms compared with healthy controls (p = .001) and hip fracture patients without depressive symptoms (p = .01) [Figure 2b]. When the above analyses were repeated with adjustment for age, sex and BMI, the results remained significant (data not shown).

To further investigate possible increases in senescent T cells in hip fracture patients other markers of senescence were examined, such as CD57 [33]. On examining CD57 expression [Figure 2c], we found significant differences in CD57^+ve^ CD4 T cells, F (2, 62) = 1.32, p = .008, η^2^ = .16 between the three groups [Figure 2d], driven by an increase in the percentage of CD57^+ve^ T cells in hip fracture patients with depressive symptoms compared with healthy controls (p = .003). Similarly, differences in frequency of circulating CD57^+ve^ CD8 T cells were seen between our three groups, F (2, 62) = 8.16, p = .001, η^2^ = .20, driven by a significant increase in the percentage of CD57^+ve^ CD8 T cells in hip fracture patients with depressive symptoms compared with healthy controls (p = .001) [Figure 2d]. When the above analyses were repeated with adjustment for age, sex and BMI, the results remained significant (data not shown).

Finally, on examining frequency of CD28^-ve^CD57^+ve^ CD4 T cells significant differences, F (2, 56) = 6.28, p = .003, η^2^ = .18 occurred [Additional file 1: Figure S1a], due to increased frequency of CD28^-ve^ CD57^+ve^ CD4 T cells in hip fracture patients with depressive symptoms compared with healthy controls, p = .002, but not on comparison with hip fracture patients without depressive symptoms, p = .13. Similarly, the frequency of CD28^-ve^ CD57^+ve^ CD8 T cells were also significantly different, F (2, 56) = 4.12, p = .02, η^2^ = .12 between the three groups [Additional file 1: Figure S1b], due to a significant increase in frequency of CD28^-ve^ CD57^+ve^ CD8 T cells in hip fracture patients with depressive symptoms compared with healthy controls, p = .02 but not in comparison with hip fracture patients without depressive symptoms, p = .49.

**Figure 3:**
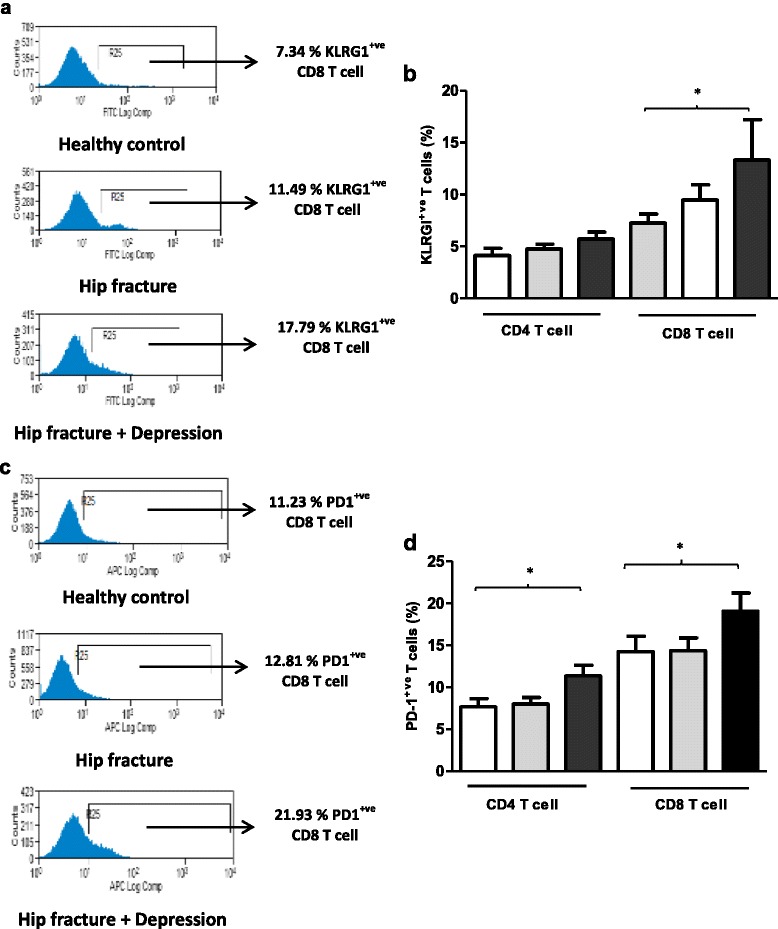
**Frequency of peripheral senescent and exhausted T lymphocytes in hip fracture patients with and without depressive symptoms versus healthy controls. (a)** Representative flow cytometry plots showing frequency of KLRG1^+ve^ CD8 T cells in a healthy control, hip fracture and a hip fracture patient with depressive symptoms. **(b)** The percentage of KLRG1^+ve^ CD4 and CD8 T cells in healthy controls: n = 23 (open bars); hip fracture patients without depression n = 19 (grey bars); and hip fracture patients with depressive symptoms n = 23 (black bars). **(c)** Representative flow cytometry plots showing frequency of PD1^+ve^ CD8 T cells in a healthy control, hip fracture and a hip fracture patient with depressive symptoms. **(d)** The percentage of PD1^+ve^ CD4 and CD8 T cells in healthy controls: n = 23 (open bars); hip fracture patients without depression n = 19 (grey bars); and hip fracture patients with depressive symptoms n = 23 (black bars). Data are mean ± SEM. *p < .05 and **p < .01.

Killer cell lectin like receptor G 1(KLRG1) has also been identified as a marker for functional senescence in T cells [34]. On examining KLRG1 expression on T cells [Figure 3a], no significant differences were reported in the frequency of KLRG1^+ve^ CD4 T cells, F (2, 55) = 2.53, p = .09, η^2^ = .08 between the three groups [Figure 3b]. However, significant differences were seen in the percentage of KLRG1^+ve^ CD8 T cells between our groups, F (2, 55) = 3.91, p = .02, η^2^ = .12, driven by an increase in hip fracture patients with depressive symptoms compared with healthy controls (p = .03).

Elevated levels of Programmed cell death (PD-1) receptor is also a hallmark of T cell dysfunction termed T cell exhaustion [35]. T cell exhaustion is characterised by a loss of effector T cell function, including cytokine production, reduced proliferation capacity and ex vivo killing [36]. Limited studies examining PD-1 with age have reported an increase in PD-1 expression on the T cells of aged mice [37,38]. In this study we report for the first time significant differences in peripheral PD1^+ve^ CD4 T cells [Figure 3c], F (2, 62) = 4.72, p = .01, η^2^ = .13 and peripheral PD1^+ve^ CD8 T cells, F (2, 62) = 3.11, p = .05, η^2^ = .09 between the three groups [Figure 3d], driven by an increase in the percentage of PD1^+ve^ CD4 T and PD1^+ve^ CD8 T cells in hip fracture patients with depressive symptoms compared with healthy controls, p = .01 and p = .05 respectively.

### Accumulation of activated T cells in hip fracture patients with depressive symptoms

**Figure 4:**
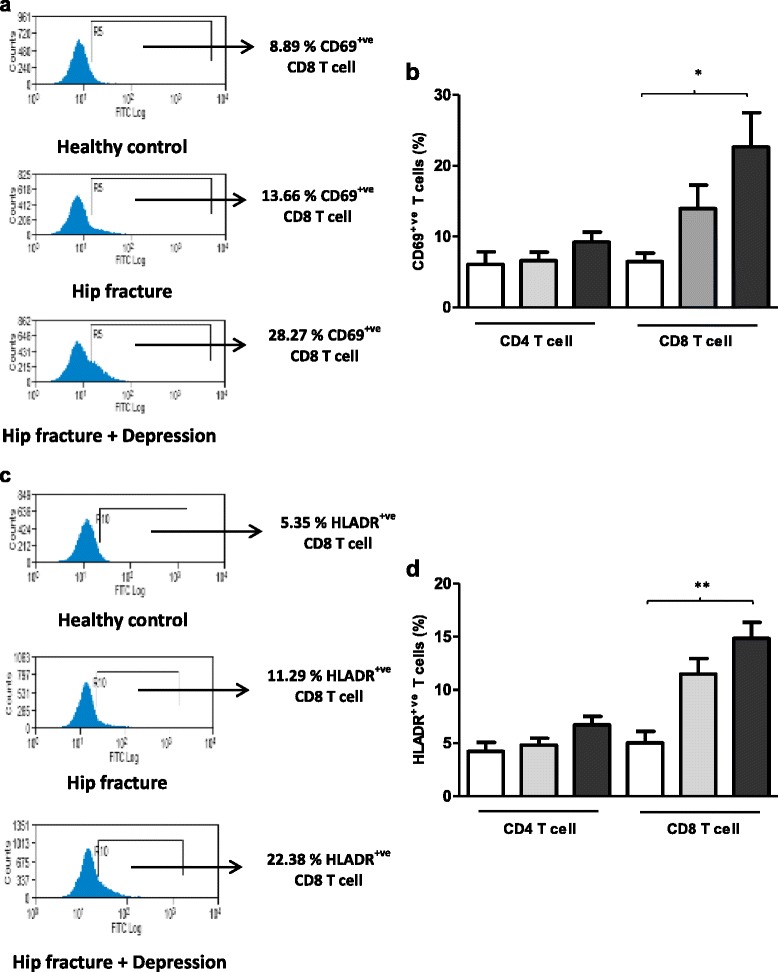
**Frequency of peripheral activated T lymphocytes in hip fracture patients with and without depressive symptoms versus healthy controls. (a)** Representative flow cytometry plots showing frequency of CD69^+ve^ CD8 T cells in a healthy control, hip fracture and a hip fracture patient with depressive symptoms. **(b)** The percentage of CD69^+ve^ CD4 and CD8 T cells in healthy controls: n = 21 (open bars); hip fracture patients without depression: n = 22 (grey bars) and hip fracture patients with depressive symptoms: n = 24 (black bars). **(c)** Representative flow cytometry plots showing frequency of HLADR^+ve^ CD8 T cells in a healthy control, hip fracture and a hip fracture patient with depressive symptoms. **(d)** The percentage of HLADR^+ve^ CD4 and CD8 T cells in healthy controls: n = 21 (open bars); hip fracture patients without depression: n = 22 (grey bars) and hip fracture patients with depressive symptoms: n = 24 (black bars). Data are mean ± SEM. *p < .01 and **p < .001.

The expression of different activation markers CD69 (early), CD25 (middle) and HLADR (late) on T cells was also examined [Figure 4a]. Firstly, on examining expression of CD69 on CD4 T cells no significant differences were observed between the three groups, F (2, 64) = 1.51, p = .003, η^2^ = .16 [Figure 4b], though significant differences were observed for CD8 T cells, F (2, 64) = 5.45, p = .006, η^2^ = .14, driven by an increase in the percentage of CD69^+ve^ CD8 T cells in hip fracture patients with depressive symptoms compared with healthy controls (p = .005).

On examining peripheral distribution of CD25^+ve^ CD4 T cells and CD25^+ve^ CD8 T cells no significant differences were found, F (2, 53) = 1.30, p = .28, η^2^ = .04 (data not shown). Similarly the frequency of HLADR expressing CD4 T cells [Figure 4c] was not different between our three groups, F (2, 60) = 2.87, p = .06, η^2^ = .08 [Figure 4d], though we did find significant differences in the frequency of peripheral HLADR^+ve^ CD8 T cells, F (2, 60) = 13.58, p < .001, η^2^ = .31 between our three groups [Figure 4d], driven by an increase in the percentage of HLADR^+ve^ CD8 T cells in hip fracture patients with depressive symptoms compared with healthy controls (p < .001). When the above analyses were repeated with adjustment for age, sex and BMI, the results still remained significant (data not shown).

### Th1/Th2/Th17 balance

**Figure 5:**
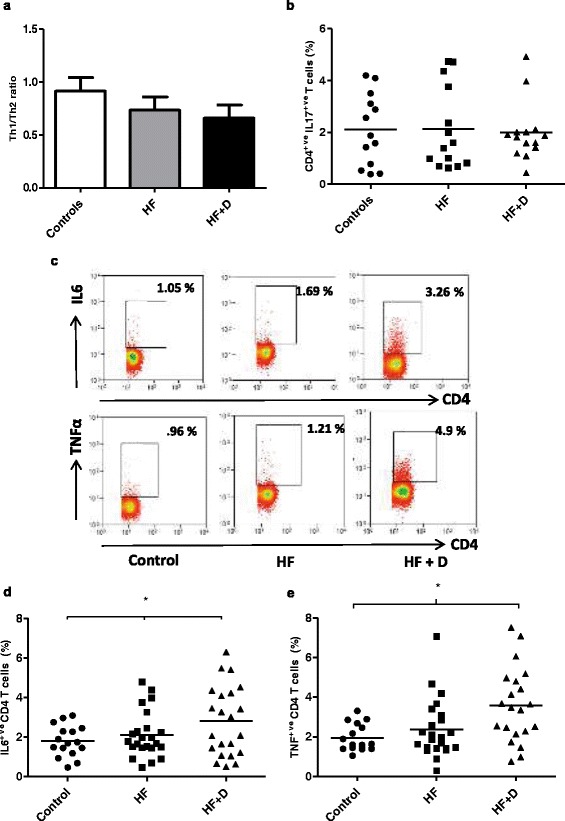
**Cytokine production by T lymphocytes in hip fracture patients. (a)** The Th1 (IFNγ^+ve^) / Th2 (IL4^+ve^) ratio, or **(b)** the percentage of IL17^+ve^ CD4 T cells in healthy controls, n = 16; hip fracture patients without depression (HF), n = 21; and hip fracture patients with depressive symptoms (HF + D), n = 21. The solid bar represents the mean value. **(c)** Representative flow cytometry plots showing frequency of IL6^+ve^ CD4 (upper panel) and TNFα^+ve^ T cells (lower panel) in a healthy control, hip fracture and a hip fracture patient with depressive symptoms. **(d)** The percentage of IL6^+ve^ CD4 T cells or **(e)** TNFα^+ve^ T cells in healthy controls, n = 16; hip fracture patients without depression (HF), n = 23; and hip fracture patients with depressive symptoms (HF + D), n = 22. The solid bar represents the mean value. The solid bar represents the mean value. *p < .05 and **p < .01.

To compare functional activity of T cells between healthy controls and hip fracture patients, the balance of IFNγ^+ve^ (Th1): IL4^+ve^ (Th2) and IL17^+ve^ (Th17) CD4 T cells was investigated post stimulation with PMA and Ionomycin. In this study, no significant differences were found in the Th1 / Th2 ratio between the three groups F (2, 59) = 1.49, p = .23, η^2^ = .04 [Figure 5a]. The frequency of IL17^+ve^ CD4 T cells post-stimulation with PMA and Ionomycin was not significantly different between our three groups F (2, 37) = .93, p = .40, η^2^ = .04 [Figure 5b]. Further, measuring circulating serum levels of IL17, revealed no significant differences between healthy controls and hip fracture patients with and without depression F (2, 63) = .36, p = .69, η^2^ = .01.

### Pro-inflammatory cytokine production by T cells in hip fracture patients

On comparing the frequency of IL6^+ve^ CD4 T cells [Figure 5c] post-stimulation with PMA and Ionomycin, significant differences were found between our three groups F (2, 57) = 3.47, p = .03, η^2^ = .10, driven by an increase in percentage of IL6^+ve^ CD4 T cells in hip fracture patients with depressive symptoms compared with healthy controls, p = .04, no significant differences were observed between hip fracture patients with and without depressive symptoms, p = .17 [Figure 5d]. The frequency of TNFα^+ve^ CD4 T cells [Figure 5c] post-stimulation with PMA and Ionomycin, showed significant differences between our three groups F (2, 58) = 3.49, p = .03, η^2^ = .10, driven by an increase in percentage of TNFα^+ve^ CD4 T cells in hip fracture patients with depressive symptoms compared with healthy controls, p = .03 [Figure 5e].

### GDS scores and T cell phenotype

**Figure 6:**
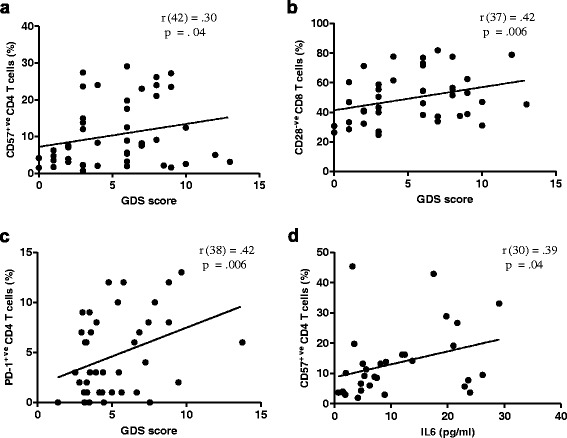
**Association between senescent T cells and depressive symptom score (GDS) and circulating pro-inflammatory cytokine IL6.** Correlation between **(a)** GDS score and circulating CD57^+ve^ CD4 T cells (n = 41), **(b)** GDS score and circulating CD28^-ve^ CD8 T cells (n = 37), **(c)** GDS and circulating PD1^+ve^ CD4 T cells (n = 38), or **(d)** IL6 levels and circulating CD57^+ve^ CD4 T cells (n = 30).

For several measures of T cell numbers or phenotype there was an effect of depression not seen in patients with hip fracture alone. To see if there was a graded association between depressive symptoms and T cells in these patients linear regression analysis was performed. In relation to senescent T cells there was an association between GDS scores and the frequency of peripheral CD57^+ve^ CD4 T cells, r (42) = .42 , p = .006 [Figure 6a], though no significant association was found for CD57^+ve^ CD8 T cells, r (42) = .15, p = .32. There was also a trend towards an accumulation of KLRG1^+ve^ T cells in hip fracture patients with higher GDS scores, but this did not reach statistical significance for the frequency of KLRG1^+ve^ CD4 T cells, r (39) = .29, p = .06, or KLRG1^+ve^ CD8 T cells, r (39) = .28, p = .07. There was no significant association between GDS scores and the frequency of peripheral CD28^-ve^ CD4 T cells, r (42) = .19, p = .22, but there was an association between GDS scores and peripheral CD28^-ve^ CD8 T cells, r (42) = .30, p = .04 [Figure 6b]. There was no significant association observed between GDS scores and the frequency of CD69^+ve^ CD8 T cells, r (46) = .17, p = .25, or HLADR^+ve^ CD8 T cells, r (42) = .16, p = .31 in hip fracture patients. There was also a significant association between GDS scores and thefrequency of PD1^+ve^ CD4 T cells, r (41) = .34, p = .02 [Figure 6c]. Interestingly, a significant association was found between circulating IL6 levels and the frequency of senescent CD57^+ve^ CD4 T cells, r (63) = .39, p = .04 [Figure 6d], such that patients with higher serum levels of IL6 had a higher frequency of circulating CD57^+ve^ CD4 T cells.

### Cortisol and T cell phenotype

Cortisol is known to induce apoptosis in T cells, therefore we determined whether there was an association between serum cortisol levels and T cell numbers. However, no association was observed between serum cortisol levels and frequency of circulating T cells (β = −.17, p = .18, ΔR^2^ = .03), absolute T cell numbers (β = −.20, p = .27, ΔR^2^ = .04), frequency of activated CD69^+ve^ CD8 T cells (β = .16, p = .22, ΔR^2^ = .02), HLADR^+ve^ CD8 T cells (β = .22, p = .11, ΔR^2^ = .04), senescent CD28^-ve^ CD8 T cells (β = − .04, p = .72, ΔR^2^ = .002), CD57^+ve^ CD8 T cells (β = − .04, p = .74, ΔR^2^ = .002), KLRG1^+ve^ CD8 T (β = .18, p = .20, ΔR^2^ = .03), PD1^+ve^ CD4 T cells (β = .07, p = .62, ΔR^2^ = .005), or PD1^+ve^ CD8 T cells (β = .28, p = .07, ΔR^2^ = .09) in hip fracture patients.

### Cytokines and T cell phenotype

A significant association was found between circulating IL6 levels and the frequency of senescent CD28^-ve^ CD57^+ve^ T cells, β = .37, p = .01, ΔR^2^ = .13, such that patients with higher serum levels of IL6 had a higher frequency of circulating CD28^-ve^ CD57^+ve^ T cells [Additional file 1: Figure S2a]. A similar association was observed between serum TNFα levels and the frequency of CD28^-ve^ CD57^+ve^ T cells, β = .44, p = .01, ΔR^2^ = .19 [Additional file 1: Figure S2b].

## Discussion

Depression is a common public health problem that has been identified by the World Health Organisation as a leading cause of disability worldwide [39]. Depression in addition to being a psychiatric disorder has also been associated with a state of immune dysfunction [40]. In the present study, we have reported that new onset depression results in both phenotypic and functional alteration in peripheral T cells in elderly hip fracture patients.

Firstly, we report a decline in T cell frequency and absolute numbers in hip fracture patients with depressive symptoms compared with healthy controls, which is in agreement with previous reports of reduced circulating T cells in depressed individuals [41,42]. Although the exact mechanism responsible has not been explored in this manuscript, a few possibilities can be proposed. Exposure to chronic stress is accompanied by increased HPA axis activity [43,44]. Glucocorticoids are known inducers of T cell apoptosis [45]. We have previously reported elevated serum cortisol levels in hip fracture patients with depressive symptoms [31]. However, there was no association between circulating T cell numbers and serum cortisol levels, suggesting that additional mechanisms may be responsible for the reduction in circulating T cells in hip fracture patients with depressive symptoms.

Evaluation of T cell subsets revealed that the proportion of CD4 and CD8 T cells and the CD4:CD8 ratio did not differ between hip fracture patients with and without depressive symptoms and healthy controls. These findings are in line with previous reports showing that the CD4:CD8 ratio was unaltered in depressed individuals [46,47]. Furthermore, hip fracture alone or in synergy with depressive symptoms did not have an effect on the naïve: memory ratio of T cells suggesting that this stress did not further exacerbate the age-related decline in naive T cells or expansion of the memory T cell pool. These findings contradict a previous study reporting an increase in memory T cells in depressed patients [48,49]. This discrepancy might be due to the differences in cell surface markers used to identify memory T cells as Maes *et al.* identified memory T cells as CD45RA^-ve^ T cells, whereas in this study both CD45RA and CCR7 were used to identify T cell subsets. In addition our patients did not suffer from chronic depression and their condition was acute in response to their injury.

It is well documented that ageing is accompanied by an accumulation of senescent T cells. On examining the additional effect of chronic stress on CD28 expression, a significant increase in the percentage of CD28^-ve^ T cells was seen, especially in CD8 T cells in hip fracture patients with depressive symptoms. Moreover, an increase in CD57^+ve^ T cells, again most marked in CD8 T cells, was also observed in hip fracture patients with depressive symptoms, which is in line with previous studies in depressed individuals [49]. Interestingly, poor mental health associated with reduced job satisfaction has also been characterised by an increase in CD57^+ve^ CD8 T cells [50]. The majority of the CD57^+ve^ T cells are also CD28^-ve^[51], therefore it is not surprising that an accumulation of CD28^-ve^CD57^+ve^ T cells was seen in hip fracture patients with depressive symptoms and this is in agreement with similar findings in depressed individuals [52]. The accumulation of these expanded T cells could reduce the T cell repertoire in hip fracture patients with new onset of depression and reduce their immune response towards novel pathogens and vaccines [53,54].

Although the exact mechanism responsible for accumulation of senescent T cells has not been explored in this manuscript, a few possibilities can be proposed. TNFα is known to induce down regulation of CD28 expression on T cells [55]. In this study we found a correlation between circulating CD28^-ve^ CD57^+ve^ T cells and serum IL6 and TNFα levels, suggesting the contribution of the pro-inflammatory environment in accumulation of senescent T cells. Further, CMV seropositivity has also been associated with senescence in the T cell compartment and accumulation of late differentiated CD28^-ve^ CD57^+ve^ T cells [56,57]. Although, CMV seropositivity has not been tested in our hip fracture patients, it is possible that there might be an association between CMV and accumulation of senescent T cells in hip fracture patients.

In addition to being an inflammatory disorder, depression is also characterised by activation of cell-mediated immunity [58]. Although we failed to report signs of monocyte activation in hip fracture patients with depressive symptoms [59], an increase in T cells expressing the activation markers CD69 and HLADR was seen in hip fracture patients with depressive symptoms. These findings are consistent with previous reports of increased activated HLADR^+ve^ T cells in depressed individuals [48,60,61]. Overall, our data point towards the existence of a state of immune activation in individuals with depressive symptoms.

Next, on exploring T cell functional properties in hip fracture patients we found no significant differences in Th1 (IFNγ)/Th2 (IL4) balance between our hip fracture patients with and without depression. Previous findings have associated depressed mood [62] with a shift in the Th1/Th2 balance towards a Th2 response, in this study we did observe a trend towards an increase in Th2 cells (IL4^+ve^ CD4 T cells) but it did not reach statistical significance. Ageing is also accompanied by a skewing towards a Th17 phenotype [63] and a similar increase in Th17 cells has been reported in patients with major depressive symptoms [64]. However, in this study no such increase in Th17 producing CD4 T cells was observed in hip fracture patients with depressive symptoms post stimulation.

Depression has been characterised as an increased inflammatory status (inflammaging) including elevated levels of pro-inflammatory cytokines, including IL6, TNFα and IL1β [65,66]. We have previously reported that elevated levels of TNFα and IL6 were seen in hip fracture patients with depressive symptoms compared with patients without depressive symptoms and healthy controls [31]. On examining pro-inflammatory cytokine production by T cells, we found that hip fracture patients with depressive symptoms had a higher capacity to produce pro-inflammatory cytokines upon stimulation. Our findings are consistent with the reports of elevated IL6 production post-stimulation in depressed patients [67]. These findings are also in line with previous reports of elevated TNFα production by peripheral blood mononuclear cells in depressed patients [68].

## Conclusions

The present study reports for the first time that development of depressive symptoms in older hip fracture patients can result in phenotypic alteration in T cells and dysregulated cytokine production. Importantly, these findings suggest that development of depressive symptoms after a hip fracture in older adults is an important driver of immune dysregulation. The clinical significance of these results still remains unexplored, but our findings support the need for preventing and treating depression and depressive symptoms to improve outcomes in older hip fracture patients.

## Methods

### Participants

101 older hip fracture patients (37 male) were recruited from five hospitals in Birmingham, UK between 2010 and 2012. Inclusion criteria were that participants had to be aged 60 years and over with a hip fracture sustained 4–6 weeks previously but with no chronic immune-related disorders e.g., cancer, diabetes, or taking any regular medications that might modify immunity, e.g., immunosuppressants, statins. Additionally patients must not have had any diagnosis of depression by a physician prior to age 50 years or be taking or have previously taken anti-depressant medication. 43 healthy older adults (26 female), were also recruited from the community as controls. The study was approved by South Birmingham Local Research Ethics Committee and all participants provided written informed consent (study ref: 09/H1203/80).

### Study design

The study design and patient demographics have been reported in full previously [69]. Briefly, the study was a prospective case–control design with three groups of older adults: hip fracture patients with or without depressive symptoms and healthy older adults. Consent was gained whilst patients were still in hospital. All patients provided a blood sample and completed questionnaires and structured interviews 4–6 weeks after hip fracture. Control participants attended the University to provide a single blood sample and complete a depression and anxiety symptoms scale questionnaire and provide basic demographic information. Blood samples were taken between 09.00 and 11.00 to minimise any effect of diurnal variations in steroid or cytokine levels. None of the participants had an acute infection at the time of blood sampling.

### Questionnaires

Standard socio-demographic and health behaviour information were taken and medications, prescription and over-the-counter, were recorded by the interviewer. The psychological status of the participant was assessed by means of standardised psychometric questionnaires. Depression was evaluated by a Geriatric Depression Scale (GDS) [70]. Depression was defined as a GDS score greater than or equal to 6. The Hospital Anxiety and Depression Scale (HADS) was also used to measure depression and anxiety [71]. The scale contains 14 items, scored from 0 (not present) to 3 (considerable), with seven assessing aspects of depression and seven assessing anxiety. Healthy control participants completed the HADS depression sub-scale in order to check that they did not have significant depressive symptoms. A cut-off of ≥8 has previously been used to indicate possible depression [72].

### Isolation of PBMCs and immunostaining for analysis of T cell subsets

PBMCs were isolated from peripheral blood by density centrifugation using Ficoll-Paque™ PLUS (GE Healthcare, Uppsala, Sweden). The blood was layered on top of 6 ml Ficoll in a 25 ml universal tube and centrifuged at 650 × g for 30 minutes with no break. Post centrifugation, the mononuclear cell layer containing PBMC was removed and added to a new universal tube and the cells were washed twice with PBS and counted using a haemocytometer. PBMCs were frozen down by re-suspending cells in freezing medium consisting of 10% DMSO (Sigma Aldrich, UK) in heat inactivated fetal calf serum (Biosera, UK) and transferring them in small aliquots into cryovials. The cryovials were transferred into a freezing container (Mr Frosty, Sigma Aldrich, UK) containing isopropanol (VWR International, UK) and the frozen cells were then stored at −80°C.

For phenotypic characterisation of T cells, isolated PBMCs were stained with a combination of fluorochrome conjugated antibodies including; anti-human CD3-PEcy7 (eBiosciences; clone:UCHT1), anti-human CD4 Alexa fluor 450 (Biolegend; clone:RPA-T4), anti-human CD8 PE (Immunotools; clone:UCHT4), anti-human CCR7 FITC (Rand D systems; clone:150503) anti-human CD45RA APC (Biolegend; clone:HI-100), anti-human CD28-APC (BD biosciences; clone:CD28.2), anti-human CD57-FITC (eBiosciences; clone:HDC57), anti-human CD69-FITC (eBiosciences; clone:FN50), anti-human CD25-APC (eBiosciences; clone:BC96), anti-human HLADR-FITC (eBiosciences; clone:H1.2 F3), anti-human KLRG1-FITC (Biolegend; clone:2 F1/KLRG1), anti-human CD279-APC (eBiosciences; clone:eBioJ105). Appropriate isotype controls were used for setting gates. Following incubation, cells were washed and resuspended in PBS for flow cytometric analysis using a Cyan™ ADP flow cytometer (Dako). T cells can be classified into four distinct subsets on the basis of expression of cell surface markers, CCR7 and CD45RA; naïve (CD45RA^+ve^ CCR7^+ve^), central memory (CD45RA^-ve^ CCR7^+ve^), effector memory (CD45RA^-ve^ CCR7^-ve^) and effector memory RA (CD45RA^-ve^ CCR7^–ve^) [32]. The gating strategy used to identify T cell subsets is shown in Figure 1c-f.

### In-vitro cultures of PBMCs to induce cytokine production by T cells

PBMC cultures were performed in complete RPMI 1640 (Sigma Aldrich, UK) containing 10% FCS (Biosera, UK) supplemented with glutamine/penicillin/streptomycin (Life Technologies, UK). PBMCs were stimulated for 4 hr with PMA (50 ng/ml; Sigma Aldrich, UK) and Ionomycin (500 ng/ml; Sigma Aldrich, UK) in the presence of Brefeldin A (10 μg/ml; Sigma Aldrich, UK). Post-stimulation, cells were washed twice with PBS and stained using anti-human CD3 PEcy7 (eBiosciences, UK; clone: UCHT1) and anti-human CD4 Alexa fluor 450 (eBiosciences, UK; clone: RPA-T4) for 20 min in the dark at 4°C. Cells were washed and fixed with 50 μl Reagent A (Fix and Perm kit, Invitrogen, UK) for 30 minutes in the dark at room temperature. Post incubation, cells were washed and re-suspended in 50 μl Reagent B (Fix and Perm kit, Invitrogen, UK) and anti-human TNFα PE (eBiosciences, UK; clone: MAb11) or anti-human IL6 APC (eBiosciences, UK; clone: MQ213A5) or anti-human IL17A Alexa fluor 647 (Biolegend, UK; clone:ebio64Dec13) or anti-human IFNγ APC (Bio legend, UK ;clone: AS.B3) and anti-human IL4 PE (eBiosciences, UK; clone:8D4-8) was added and samples were incubated in the dark at room temperature for 30 minutes. After washing the cells were analysed on a Cyan™ ADP (Dako Ltd, UK).

### Serum cytokines and cortisol

Serum IL-6 and TNFα were measured using multiplex technology and a commercial 5-plex kit (BioRad, Hemel Hempstead, UK). Serum cortisol was measured using a commercial ELISA kit (IBL international, Hamburg, Germany).

### Statistical analysis

Univariate ANOVA with least significant difference post-hoc tests were used to assess differences between the three groups (hip fracture with depressive symptoms, hip fracture without depressive symptoms, and healthy controls). Where demographic variables differed significantly between the three groups, analyses were rerun adjusting for these variables using ANCOVA. Pearson’s correlations were used to examine associations between depression score/serum hormone levels/circulating cytokine levels and T cell phenotype and cytokine production.

## Additional file

Additional file 1: Figure S1. CD28^-ve^ CD57^+ve^ T lymphocytes in hip fracture patients. Percentage of (a) CD28^-ve^CD57^+ve^ CD4 T cells, or (b) CD28^-ve^CD57^+ve^ CD8 T cells in healthy controls (n = 23), hip fracture patients without depressive symptoms (HF; n = 19) and hip fracture patients without depressive symptoms (HF + D; n = 17). The solid bar represents the mean value. *p <.05. **Figure S2.** Association between circulating pro-inflammatory cytokines and senescent T cells. Correlation between (a) serum IL6 levels and the frequency of circulating CD28^-ve^ CD57^+ve^ T cells (n = 44) (b) serum TNFα levels and frequency of circulating CD28^-ve^ CD57^+ve^ T cells (n = 29).
