# Supplementary figures and images for: Depressive symptoms post hip fracture in older adults are associated with phenotypic and functional alterations in T cells
Source: Immun Ageing. 2014 Dec 16;11:25. doi: 10.1186/s12979-014-0025-5 (PMC4307912; doi:10.1186/s12979-014-0025-5)

**a**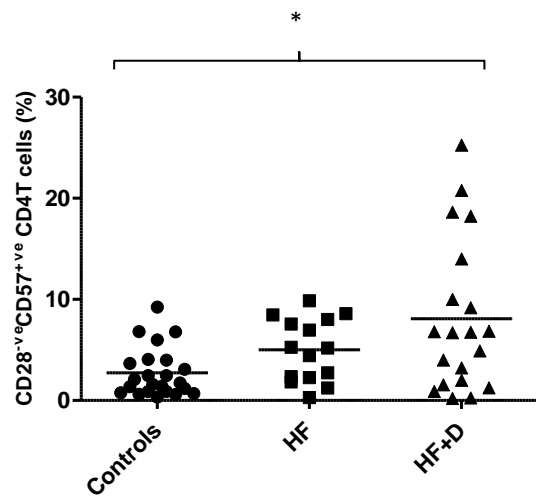**b**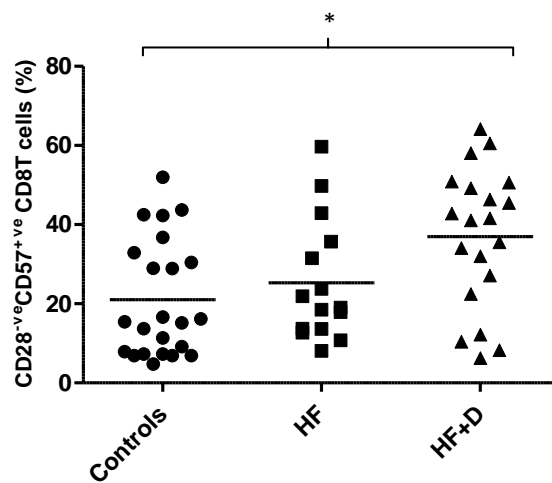

Supplementary Figure 1

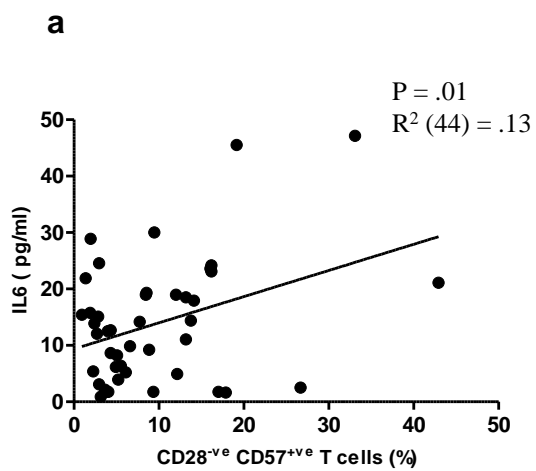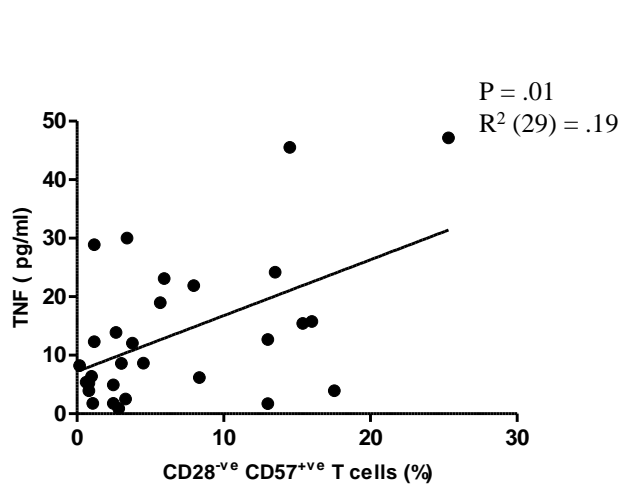

Supplementary Figure 2

Supplement: Additional file 1: Figure S1. — CD28-ve CD57+ve T lymphocytes in hip fracture patients. Percentage of (a) CD28-veCD57+ve CD4 T cells, or (b) CD28-veCD57+ve CD8 T cells in healthy controls (n = 23), hip fracture patients without depressive symptoms (HF; n = 19) and hip fracture patients without depressive symptoms (HF + D; n = 17). The solid bar represents the mean value. *p <.05. Figure S2. Association between circulating pro-inflammatory cytokines and senescent T cells. Correlation between (a) serum IL6 levels and the frequency of circulating CD28-ve CD57+ve T cells (n = 44) (b) serum TNFα levels and frequency of circulating CD28-ve CD57+ve T cells (n = 29). [file 12979_2014_25_MOESM1_ESM.pdf]
